# Replacement of added trace minerals (zinc, copper, iron, and manganese) by a consensus bacterial 6-phytase variant in weaned pigs fed an all-vegetable diet

**DOI:** 10.1093/jas/skaf419

**Published:** 2025-12-05

**Authors:** Leon Marchal, Georg Dusel, Katharina Schuh-Von Graevenitz, Deepak E Velayudhan, Ester Vinyeta, Yueming Dersjant-Li

**Affiliations:** Danisco Animal Nutrition & Health, IFF, Oegstgeest 2342 BH, The Netherlands; Department of Life Sciences and Engineering, University of Applied Sciences Bingen, Bingen am Rhein 55411, Germany; Department of Life Sciences and Engineering, University of Applied Sciences Bingen, Bingen am Rhein 55411, Germany; Danisco Animal Nutrition & Health, IFF, Oegstgeest 2342 BH, The Netherlands; Danisco Animal Nutrition & Health, IFF, Oegstgeest 2342 BH, The Netherlands; Danisco Animal Nutrition & Health, IFF, Oegstgeest 2342 BH, The Netherlands

**Keywords:** bioavailability, phytase, trace minerals, weaned pigs

## Abstract

This experiment evaluated whether a consensus bacterial 6-phytase variant (PhyG) could totally replace the effect of added trace minerals (**TM**; Zn, Cu, Fe, and Mn) in an all-vegetable diet, in weaned pigs. A total of 144 DanBred × Pietrain weaned pigs (28 d of age, body weight 7.0 ± 0.44 kg) were assigned to floor pens (12 pens/treatment; 4 pigs/pen; 2 females, 2 castrated males). Diets were based on corn, wheat, barley and soybean meal, fed in 2 phases (starter I: day 1 to 14 and starter II: day 14 to 42) and provided ad libitum. Treatment diets comprised: 1) a negative control (**NC1**) diet formulated without added TM but otherwise nutritionally adequate; 2) a positive control (**PC**), comprising the NC1 supplemented with Zn, Cu, Fe, and Mn at 120, 80, 96, and 80 mg/kg, respectively, and; 3) the NC1 reduced in Ca and digestible P to account for the expected contribution of PhyG which was supplemented at 1,500 phytase units (**FTU**)/kg during starter I and 1,000 FTU/kg during starter II (**NC2+PhyG**). Growth performance was monitored over 42 d, and on day 42 blood, liver and femur bone samples were collected from 1 pig/pen for TM analysis. Data were analyzed by one-way ANOVA and treatment means were separated by Tukey’s HSD test. Pen was the experimental unit. Final (day 42) body weight and overall average daily gain were reduced in NC1 compared with PC (−2.04 kg/pig and −48.8 g/pig/day, respectively; *P *< 0.05) and increased (*P *< 0.05) in NC2+PhyG compared with NC, to levels not different from the PC. Zinc in bone ash at day 42 was increased in the PC compared with NC1 (+25.7 mg/kg, respectively; *P *< 0.05) and further increased in NC2+PhyG vs. NC1 or PC (+49.9 and +24.2 mg/kg, respectively), whereas Fe in bone was increased in NC2+PhyG vs PC (+35.4 mg/kg; *P *< 0.05). The results indicate that PhyG supplementation at the applied doses maintained growth performance and tissue TM concentrations at levels comparable to those achieved by the TM-supplemented diet. The findings suggest that the enzyme could replace supplemental Zn and may also support a reduction in the level of supplemental Cu, Fe and Mn in piglet diets, depending on the content of these TM in the basal diet.

## Teaser

This experiment has demonstrated that a consensus bacterial 6-phytase variant, when added to an all-vegetable diet containing no added trace minerals at a level of 1,500 phytase units per kilogram of diet during starter I phase and 1,000 FTU/kg during starter II phase, can maintain growth performance and bone mineralization at levels not different to those achieved by conventional supplementation of zinc, copper, iron, and manganese, in weaned pigs.

## Introduction

Trace minerals (**TM**) are critical for swine health. They perform vital functions as co-factors for enzymes, regulators of oxidative stress and blood homeostasis, and are important components of bone and muscle tissue (Sampath et al., 2023). Trace mineral bioavailability in conventional feedstuffs is low. Hence, to ensure requirements are met, Zn, Cu, Fe, and Mn are commonly supplemented in piglet diets. However, amounts of supplemented TM vary considerably among feed producers (Flohr et al., 2016), across geographical regions (Dalto and da Silva, 2020) and by pig age (higher in feed for young animals than in older animals; Flohr et al., 2016). In many cases, supplementation may be far in excess of animal requirements, or at least in excess of recommendations such as those of the NRC (Flohr et al., 2016; Faccin et al., 2023). Any excess or unused TM is excreted. Excretion of TM is undesirable both on cost and environmental sustainability grounds. Regulatory authorities in the EU have issued strict maxima in feed for certain TM including Zn and Cu, in order to reduce loads on the environment (EFSA, 2014; European Commission, 2018).

One of the main reasons for the low bioavailability of native TM in basal diets containing vegetable ingredients is the binding of positively charged TM ions with negatively charged phytate (phytic acid) in the digesta of pigs (Humer et al., 2015). It has been observed that when dietary Zn is high (>1,000 mg/kg), this reduces the digestibility of P, due to binding with phytate (Labarre et al., 2025). Liu et al. (2014) estimated the apparent total tract digestibility (**ATTD**) of Zn, Cu, Fe, and Mn in a corn-soybean meal-based grower pig diet to be 33.9%, 37.1%, 29.1%, and 29.5%, respectively. This means that even if TM levels in the basal diet are close to requirements, a substantial portion of ingested TM will be undigestible and excreted (Liu et al., 2014). One strategy for improving TM utilization and thereby reducing their excretion is to improve the availability of TM within feed raw materials. Microbial phytase is routinely added to pig diets for the primary purpose of improving the digestibility of P (Selle and Ravindran, 2008) but it can also increase the digestibility of Ca and other macrominerals as well as amino acids (**AA**), starch and energy (She et al., 2018; Espinosa et al., 2022; Velayudhan et al., 2025). The mode of action of PhyG in hydrolyzing plant-derived phytate to release inorganic phosphate reduces the abundance of phytate in the digesta. This reduces the availability of phytate to bind with TM ions which occurs at both low pH such as that in the stomach (pH 2.5 to 3.5; Dersjant-Li et al., 2015) and at higher pH such as that in the small intestine (pH 4.0 to 6.5) where TM-phytate complexes become insoluble and hard to digest (Selle et al., 2009). Therefore, a reduction in phytate abundance may reasonably be expected to increase the availability of unbound TM ions for digestion and absorption. Indeed, several studies have shown improved digestibility and retention of TM in pigs fed diets supplemented with phytase (Peter et al., 2001; Revy et al., 2004, 2006; She et al., 2018; Czech et al., 2022). In the case of Zn, the sparing effect has been quantified for some individual phytases according to dose (Revy et al., 2004, 2006; Bikker et al., 2012) but also appears to be affected by the level of Zn in the diet; when the Zn supplementation level is high, the beneficial effect of phytase on digestible Zn could be reduced (Labarre et al., 2025).

The majority of the existing studies on phytase and TM bioavailability have been in grower and finisher pigs in which TM requirements are lower than in newly weaned pigs (and therefore the capacity of phytase to replace TM may be greater), or have compared the effect of phytase with supplementation of only a single TM rather than multiple TM. Of interest to producers and feed formulation scientists is to know whether a supplemental phytase could totally replace commercial levels of multiple commonly supplemented TM in the diet.

This study tested the hypothesis that PhyG phytase added to an all-vegetable diet at 1,500 phytase units (**FTU**)/kg during starter I phase and 1,000 FTU/kg during starter II phase, would improve TM availability and utilization in weaned pigs, thereby maintaining growth performance and tissue TM concentrations at levels comparable to those achieved by a similar diet supplemented with 120, 80, 96, and 80 mg/kg of Zn, Cu, Fe, and Mn, respectively, but without PhyG.

## Materials and Methods

This study was conducted at the University of Applied Sciences Bingen in Germany. The research was conducted in accordance with animal welfare practice guidelines in Germany, and with European Directive 2008/120/EC laying down minimum standards for the protection of pigs (European Council, 2008). As the pigs in the study were kept in conventional pens and tissues not removed until after slaughter, specific approval by the local authority was not required.

### Experimental design, pigs, and housing

The experiment was performed as a randomized complete block design with 3 treatments. The location of pens within the barn was the blocking factor. A total of 144 DanBred × Pietrain weaned pigs [28 d of age, initial body weight (**BW**) 7.0_** ± **_0.44 kg] were randomly assigned to floor pens with 4 pigs per pen (2 females, 2 castrated males) and 12 replicate pens per treatment. Pens were allocated to treatments so that each treatment contained pigs of approximately the same initial average BW. Pens contained slatted floors and were located in an environmentally controlled animal house in which temperature was maintained at 30 _**°**_C during the first 7 d and thereafter gradually reduced to 24_** °**_C by day 42. The lighting regime was LD 9:15 h, supplemented by natural daylight.

### Diets

Treatment diets were based on corn, wheat, barley, and soybean meal, with added rapeseed meal. Diets were formulated in two feeding phases: day 1 to 14 (starter I) and day 14 to 42 (starter II). A summary of the differences between the 3 treatment diets is presented in Table 1. The 3 treatment diets comprised: 1**)** A negative control (**NC1**), formulated without supplemental Zn, Cu, Fe, or Mn but otherwise providing adequate nutrients for growing piglets according to the recommendations in Germany (GfE, 2006); 2**)** a positive control (**PC**) based on the NC but supplemented with Zn (from ZnSO_4_), Cu (CuSO_4_), Fe (from Fe SO_4_), and Mn (from MnSO_4_) at 120, 80, 96, and 80 mg per kilogram of diet, respectively, these levels having been selected to fall within the range of commonly supplemented levels for these TM in commercial diets for young pigs in Germany at the time the experiment was conducted, and; 3**)** a diet reduced in Ca and in apparent total tract digestible P by 0.12% and 0.19% points, respectively, vs NC1, to account for the expected contributions of PhyG, which was supplemented at 1,500 FTU/kg during starter I and 1,000 FTU/kg during starter II (**NC2+PhyG**). PhyG was a consensus bacterial 6-phytase variant produced in *Trichoderma reesei* (Axtra PHY GOLD, Danisco Animal Nutrition & Health, IFF, Oegstgeest, The Netherlands). The full ingredient and calculated nutrient composition of the treatment diets is presented in Table 2.

**Table 1.** Treatment details

Treatment	Description	PhyG, FTU/kg feed		Added trace minerals, mg/kg feed during all phases^1^			
		Starter (day 1 to 14)	Starter II (day 14 to 42)	Zn	Cu	Fe	Mn
**1**	NC1^2^	-	-	-	-	-	-
**2**	PC	-	-	120	80	96	80
**3**	NC2+PhyG^3^	1,500	1,000	-	-	-	-

1 Zn, from ZnSO_4_; Cu, from CuSO_4_; Fe, from Fe SO_4_; Mn, from MnSO_4_.

2 Formulated to meet the nutrient requirements for piglets applicable in Germany (GfE, 2006) but without trace mineral supplementation.

3 Reduced in Ca and digestible P vs. NC1, to account for the expected contributions of the enzyme. The reduction in digestible P meant that this diet contained no monocalcium phosphate (MCP).

**Table 2.** Ingredient and calculated nutrient composition of the basal diets

Item	Treatment^1^							
	Starter I, (day 1 to 14)					Starter II (day 14 to 42)		
	NC1	PC		NC2+PhyG		NC1^1^	PC	NC2+PhyG
**Ingredients, % as-fed**								
** Corn**	40.0	40.0		40.0		43.0	43.0	43.0
** Wheat**	11.1	11.1		12.0		11.3	11.3	11.6
** Barley**	12.0	12.0		12.0		15.0	15.0	15.0
** Soybean meal**	22.0	22.0		22.0		18.0	18.0	18.0
** Rapeseed meal**	3.00	3.00		3.00		6.0	6.0	6.0
** Whey powder/whey fat concentrate**	5.00	5.00		5.00		-	-	-
** Vegetable oil**	2.60	2.60		2.50		2.90	2.90	2.90
** L-Lys HCl**	0.60	0.60		0.60		0.61	0.61	0.61
** DL-Met**	0.20	0.20		0.20		0.17	0.17	0.17
** L-Thr**	0.20	0.20		0.20		0.17	0.17	0.17
** L-Trp**	0.05	0.05		0.05		0.05	0.05	0.05
** L-Val**	0.05	0.05		0.05		0.05	0.05	0.05
** Salt**	0.40	0.40		0.40		0.40	0.40	0.40
** Limestone**	0.80	0.80		1.00		0.90	0.90	1.10
** Monocalcium phosphate**	1.00	1.00		-		0.50	0.50	-
** Vitamin-mineral premix – with TM^2^**	-	1.00		-		-	1.00	-
** Vitamin-mineral premix – without TM^3^**	1.00	-		1.00		1.00	-	1.00
**PhyG, FTU/kg^4^**	-	-		1,500		-	-	1,000
**Total**	100	100		100		100	100	100
**Chemical composition, % as-is (unless otherwise stated)**								
** Metabolizable energy, kcal/kg**	3,298	3,298		3,298		3,274	3,274	3,274
** Net energy, kcal/kg**	2,510	2,510		2,510		2,486	2,486	2,486
** Ash**	5.83	5.83		5.03		5.6	5.6	5.7
** Crude protein**	18.0	18.0		18.0		17.0	17.0	17.0
** Crude fat**	5.28	5.28		5.29		5.8	5.8	5.7
** Crude fiber**	3.05	3.05		3.07		3.3	3.3	3.3
** Starch**	40.1	40.1		40.6		43.7	43.7	44.2
** SID Lys**	1.24	1.24		1.24		1.15	1.15	1.15
** SID Met**	0.46	0.46		0.46		0.42	0.42	0.42
** SID Met+Cys**	0.73	0.73		0.73		0.69	0.69	0.69
** SID Thr**	0.76	0.76		0.76		0.68	0.68	0.68
** SID Trp**	0.22	0.22		0.22		0.20	0.20	0.20
** SID Val**	0.78	0.78		0.78		0.72	0.72	0.72
** Ca**	0.72	0.72		0.60		0.72	0.72	0.61
** P**	0.60	0.60		0.38		0.60	0.60	0.37
** ATTD P**	0.33	0.33		0.14		0.33	0.33	0.12
** Phytate-P**	0.23	0.25		0.25		0.23	0.25	0.25
** Na**	0.20	0.20		0.20		0.20	0.20	0.20
** Zn, mg/kg**	27	132		27		28	132	27
** Cu, mg/kg**	5	78		5		5	78	5
** Fe, mg/kg**	177	263		122		154	240	127
** Mn, mg/kg**	23	115		22		24	116	23

1 Treatment details are provided in Table 1.

2 A commercially available premix that provided per kilogram of feed: 16,000 IU vitamin A, 2,000 IU vitamin D3; 100 mg vitamin E; 4 mg vitamin K_3_; 97.5 mg vitamin C; 250 mg vitamin H; 2 mg vitamin B1; 8 mg vitamin B_2_; 20 mg Ca-D-pantothenic acid; 30 mg niacinamide; 6 mg vitamin B_6_; 2 mg folic acid; 40 mg vitamin B_12_; 500 mg choline chloride; 96 mg Fe from sulfate; 2.5 mg iodine, 80 mg Cu from sulfate; 80 mg Mn from sulfate; 120 mg Zn from sulfate; 0.4 mg Se from Na-Selenium.

3 Premix composition as in footnote 3 but without any Zn, Cu, Fe or Mn.

4 A consensus bacterial 6-phytase variant produced in *Trichoderma reesei* (Danisco Animal Nutrition & Health, IFF, Oegstgeest, The Netherlands).

ATTD, apparent total tract digestible; SID, standardized ileal digestible.

The NC1and NC2 diets were each prepared from a bulk batch of ingredients containing the common, constant, ingredients. The NC1 diet was then split into 2 sub-batches to which the specialized vitamin-mineral premix (containing supplemental TM or not, according to treatment) was added, according to treatment. The NC2 diet was supplemented with PhyG by premixing the PhyG with a small portion of the final diet. All final diets were thoroughly mixed to ensure an even distribution of ingredients and then pelleted (pelleting temperature 70 _**°**_C). Diets were provided ad libitum via an automatic feeder and water was freely available for the duration of the experiment.

### Measurements and sampling

Samples of all final diets were collected and analyzed for proximate nutrients and PhyG activity. Individual pig BW was measured at the start and the end of each phase. Pigs were monitored twice daily for health and mortality and any dead animals removed and weighed. Feed disappearance was determined at the end of each phase and used to calculate average daily feed intake (**ADFI**) per pig per phase, corrected for mortality. The gain: feed per phase and overall was calculated as BW gain per pen, including BW of dead or removed pigs, divided by the amount of feed consumed per pen.

On the last day of the experiment (day 42), pigs were slaughtered by electrical stunning and blood samples (9 mL, in duplicate) were taken immediately from the jugular vein of 1 pig (castrated male) per pen for the analysis of TM. The right femur and samples of liver tissue taken from the lower posterior double segment (same location for each sample) were collected from the same pigs and used to analyze bone TM content and liver TM content. Serum was harvested from whole blood samples after 30 min of clotting time by centrifugation at 2,000 × g for 10 min at room temperature. Serum was stored at −20 _**°**_C until analysis. Liver samples (50 to 100 g) were weighed, cut into smaller pieces, kept on ice during transport to the analytical laboratory and then frozen at −20 _**°**_C until analysis. Bones were cleaned of muscle and fat residues, autoclaved to remove meat residues and periosteum and oven dried at 60 _**°**_C for 24 h followed by incineration at 550 _**°**_C for 4 h, prior to analysis.

### Chemical analysis

Diet samples (200 to 500 g) were analyzed for dry matter (**DM**), ash, crude protein (**CP**), crude fat, crude fiber (**CF**), acid binding capacity (ABC), Ca, P, Zn, Cu, Fe, and Mn. Analyses were performed in duplicate and average of results taken. Trace minerals, Ca and P were analyzed by NutriControl analytical solutions (Veghel, The Netherlands) whereas all other nutrients were analyzed by LUFA Nord-West (Oldenburg, Germany). Diet samples were ground to pass through a 1 mm mesh screen before analysis. Dry matter and ash were analyzed according to method VDLUFA MB Bd.III, 3.1, CP was analyzed by method VDLUFA MB Bd.III, 4.1.2, crude fat was analyzed according to method VDLUFA MB Bd.III, 5.1.1, CF was analyzed by method VDLUFA MB Bd.III, 6.1.1 (all Verband Deutscher Landwirtschaftlicher Untersuchungs- und Forschungsanstalten (VDLUFA) et al., 1976–2004). Phosphorus and Ca were analyzed according to method DIN EN ISO 11885:2009-09 (DIN EN ISO, 2009). Zinc, Cu, Fe, and Mn were analyzed using validated and accredited methods based on inductively coupled plasma-optical emission spectroscopy (**ICP-OES**). The performance characteristics of the methods adhered to NEN-EN 15510:2017 (NEN-EN, 2017). Acid binding capacity was measured using the method reported by (Proh**á**szka and Baron 1980). Phytase activities in feed were analyzed by Danisco Animal Nutrition Research Centre (Brabrand, Denmark) according to a modified version of the 2000.12 AOAC method (Engelen et al., 2001), where one FTU was defined as the quantity of enzyme that released 1_** µ**_mol of inorganic orthophosphate from a 0.0051 mol/L sodium phytate substrate per minute at pH 5.5 at 37 _**°**_C. Phytate was analyzed according to the high-performance liquid chromatography method described by Christensen et al. (2020), modified from the method of Skoglund et al. (1998).

Zinc, Cu, Fe, and Mn in serum were analyzed by Labokiln N.V. (Hoensbroek, The Netherlands) using commercially available test kits. Zinc, Cu and Fe were analyzed photometrically using a Cobas 8000 modular analyzer with C701 photometric measuring unit (Roche Diagnostics Ltd), and Mn was analyzed by atomic absorption spectrometry (**AAS**) using a ZEEnit 650 P spectrometer (Analytik Jena GmbH). Zinc, Cu, Fe, and Mn in fresh-frozen liver samples and dried bone samples were analyzed by NutriControl analytical solutions (Veghel, The Netherlands) using the same ICP-OES methods described above for the analysis of these TM in diet samples.

### Statistical analysis

Pen was the experimental unit for all statistical analyses. Data were analyzed by one-way analysis of variance (**ANOVA**) to determine differences in response among treatments. Treatment was included as a fixed effect and block as random effect in this analysis. Means separation was achieved using Tukey’s HSD test. All analyses were conducted in JMP version 16.0 (JMP, 2021). A *P*-value of < 0.05 was considered statistically significant, whereas 0.1 > *P *> 0.05 was considered a statistical tendency.

## Results

### Analyzed nutrients and phytase activity

Analyzed nutrients, TM and phytase activities in the experimental diets are presented in Table 3. Nutrient levels matched well with the formulated levels. Acid binding capacity was slightly lower in NC1 compared to PC and NC2+PhyG. Trace mineral levels were variable but consistently within 35% of target values except for Mn in the PC diets which was low (54% and 56% below target in starter I and II diets, respectively). Despite this, Mn was higher in PC than NC1 or NC2+PhyG diets, as intended. Phytase activities in the NC2+PhyG diet confirmed the presence of supplemental PhyG and were comparatively low in the unsupplemented NC1 and PC diets (_**∼**_300 to 500 FTU/kg).

**Table 3.** Analyzed nutrients (%), trace minerals (mg/kg), and PhyG activities (FTU/kg) in the experimental diets

Item	Phase	Treatment^1^		
		NC1	PC	NC2+PhyG
**Dry matter**	Starter I	85.8	85.7	86.7
	Starter II	84.9	86.1	84.8
**Ash**	Starter I	5.2	5.2	5.3
	Starter II	6.9	6.8	6.4
**Crude Protein**	Starter I	17.1	17.5	17.5
	Starter II	16.5	16.7	16.4
**Crude Fat**	Starter I	5.2	4.5	4.4
	Starter II	4.2	4.8	4.4
**Crude Fiber**	Starter I	3.4	3.4	4.3
	Starter II	3.5	3.7	3.9
**Ca**	Starter I	0.82	0.82	0.73
	Starter II	0.86	0.85	0.77
**P**	Starter I	0.65	0.64	0.43
	Starter II	0.64	0.65	0.41
**Phytate-P^2^**	Starter I	0.24	n.a.	n.a.
	Starter II	0.24	n.a.	n.a.
**Zinc**	Starter I	42.5	130.5	39.5
	Starter II	42.0	138.5	44.0
**Copper**	Starter I	5.7	58.0	5.6
	Starter II	6.0	66.5	5.6
**Iron**	Starter I	216	284	162
	Starter II	228	282	168
**Manganese**	Starter I	34.0	51.0	30.0
	Starter II	32.0	53.5	29.5
**ABC-4^3^**	Starter I	485	500	539
	Starter II	505	517	510
**PhyG**	Starter I	324	347	1,778 (1,443)^4^
	Starter II	437	517	1,960 (1,483)^4^

1 Treatment details are provided in Table 1.

2 Only analyzed in NC1 but expected to be similar in NC2+PhyG as the same basal diet was used for PC and NC2.

3 Acid-binding capacity measured at pH 4, meq HCL/kg.

4 Values in parentheses are after subtracting the basal PhyG level (average of PC and NC1 diets).

n.a., not analyzed.

### Growth performance

The effects of treatment on growth performance are shown in Table 4. Mortality was low (< 3%) across all treatments and did not differ by treatment (data not shown). Feed intake and FCR did not differ among treatments during either phase or overall. During starter I (day 1 to 14), ADG and day 14 BW were reduced in pigs fed the NC1 compared with the PC (−0.33 g/pig and −0.47 kg/pig, respectively; *P *< 0.05) and not different in pigs fed NC2+PhyG compared with those fed the PC. During starter II (day 14 to 42), ADG differed by treatment (ANOVA *P *= 0.038), but Tukey’s Test did not identify any significant differences between the means. For the overall period (day 1 to 42), ADG was increased (*P *< 0.05) in PC and NC2+PhyG compared with NC1 (+48.7 and +43.5 g/pig, respectively), with no significant difference in the degree of increase between the 2 treatments. Feed intake during the overall period also tended to be increased (*P *= 0.09) in PC and NC2+PhyG compared with NC1. Final BW at day 42 was higher (*P *< 0.05) in PC and NC2+PhyG compared with NC1 (+2.04 and +1.83 kg/pig, respectively) and did not differ between these two treatments.

**Table 4.** Effect of dietary supplementation with trace minerals or PhyG on growth performance

Item	Treatments^1^			SEM	*P*-value
	NC1	PC	NC2+PhyG		
**Starter I (day 1 to 14)**					
** d 14 BW (kg/pig)**	9.53^b^	10.00^a^	9.89^ab^	0.112	0.024
** ADG (g/pig)**	180.5^b^	213.9^a^	206.2^ab^	8.03	0.024
** ADFI (g/pig)**	252.1	282.7	282.6	12.95	0.173
** Gain:feed (g:g)**	0.716	0.751	0.733	0.014	0.259
**Starter II (day 14 to 42)**					
** d 42 BW (kg/pig)**	24.48^b^	26.52^a^	26.31^a^	0.733	0.016
** ADG (g/pig)^2^**	711.9	786.7	780.9	32.64	0.038
** ADFI (g/pig)**	1,150.8	1,256.6	1,259.7	41.67	0.125
** Gain:feed (g:g)**	0.618	0.624	0.618	0.008	0.816
**Overall (day 1 to 42)**					
** ADG (g/pig)**	416.1^b^	464.8^a^	459.6^a^	17.46	0.016
** ADFI (g/pig)**	659.4	722.5	724.0	23.03	0.092
** Gain:feed (g:g)**	0.631	0.641	0.633	0.008	0.654

1 Treatment details are provided in Table 1.

2 ANOVA *P* value <0.05 but Tukey test did not show different superscript

ADFI, average daily feed intake.

### Bone mineralization

The effect of treatment on bone mineralization is shown in Table 5 (Concentrations of Mn in bone were below the limit of detection, hence not shown). Phosphorus levels in bone were similar across treatments, whereas Ca levels were higher (*P *< 0.05) in pigs fed NC2+PhyG than pigs fed NC1 or PC (+5.0 g/kg and +3.9 g/kg, respectively). Copper concentrations did not differ among treatments, whereas Zn in bone was increased (*P *< 0.05) in the PC compared with NC1. The concentration of Zn was further increased (*P *< 0.05) in NC2+PhyG compared with the PC or NC1 (+49.9 and +24.2 mg/kg, respectively). The concentration of Fe in bone was increased (*P *< 0.05) in NC2+PhyG vs. the PC (+35.4 mg/kg) and numerically higher (*P *> 0.05) in NC2+PhyG vs. the NC1 (195.4 vs. 173.4 mg/kg).

**Table 5.** Effect of dietary supplementation with trace minerals or PhyG on femur trace mineral and Ca and P content at day 42

Item^1^	Treatments^2^			SEM	*P*-value
	NC1	PC	NC2+PhyG		
**Cu, mg/kg**	2.2	2.3	2.3	0.22	0.890
**Zn, mg/kg**	185.5^c^	211.2^b^	235.4^a^	5.04	<0.001
**Fe, mg/kg**	173.4^ab^	160.0^b^	195.4^a^	10.07	0.056
**Ca, g/kg**	363.5^b^	364.6^b^	368.5^a^	1.08	0.010
**P, g/kg**	169.6	170.2	165.4	1.88	0.160

1 For Mn it is under detection limit, therefore no values are reported.

2 Treatment details are provided in Table 1.

### Trace minerals in blood and liver

Concentrations of TM in serum and liver tissue did not differ significantly among treatments (Tables 6 and 7).

**Table 6.** Effect of dietary supplementation with trace minerals or PhyG on serum trace mineral content at day 42

Item	Treatments^1^			SEM	*P*-value
	NC1	PC	NC2+PhyG		
**Cu, mg/L**	1.57	1.52	1.69	0.135	0.667
**Zn, mg/L**	2.53	2.50	3.09	0.251	0.188
**Fe, mg/L**	179.5	169.3	163.7	12.69	0.677
**Mn, µg/L**	15.8	14.9	19.6	1.81	0.167
**Se, µg/L**	100	100	107	6.14	0.643

1 Treatment details are provided in Table 1.

**Table 7.** Effect of dietary supplementation with trace minerals or PhyG on liver trace mineral and mineral content at day 42

Item	Treatments^1^			SEM	*P*-value
	NC1	PC	NC2+PhyG		
**Cu, mg/kg**	121.6	170.2	121.7	12.36	0.174
**Zn, mg/kg**	90.3	78.8	97.1	5.132	0.340
**Fe, mg/kg**	60.6	59.6	62.2	5.179	0.980
**Mn, mg/kg**	3.04	2.92	2.86	0.074	0.621
**Mg, g/kg**	0.206	0.207	0.205	0.002	0.889
**Ca, g/kg**	0.066	0.058	0.063	0.003	0.526
**P, g/kg**	3.42	3.42	3.47	0.031	0.773

1 Treatment details are provided in Table 1.

## Discussion

The NC2+PhyG treatment was formulated with reduction of total Ca and digestible P based on the expected contribution of phytase, which could potentially alter the dietary acid-binding capacity (ABC-4). However, the reduction of total Ca and dig P was achieved by reducing the inclusion of MCP and increasing the inclusion of limestone, which could potentially increase the ABC-4. Indeed the ABC-4 was slightly higher in starter I but comparable in starter II when comparing NC2+PhyG with PC, the NC diet showed lower ABC-4 which could be related to lower TM content. The analyzed ABC-4 was in a range of 485 to 539 meq HCL/kg, this difference is not expected to have major impact on TM bioavailability.

The content of TM in the basal, unsupplemented, NC diet was insufficient to support optimal weight gain during the 42-d experimental period compared with the response of pigs fed the TM-supplemented diet (PC). This indicates that the availability of TM in the NC was insufficient to fully meet requirements for growth. These results are in agreement with those of Shelton et al. (2005) who observed reduced growth performance and bone mineral content when TM were removed from the diet of nursery pigs. It is well known that young growing pigs have high TM requirements and are very sensitive to TM insufficiency (Underwood and Suttle, 1999), whereas older, grower-finisher, pigs can tolerate TM withdrawal without impairment of growth (Kim et al., 1997; McGlone, 2000; Peter et al., 2001). The content of Zn in the basal diet was well below NRC (2012) recommendations (by 58 mg/kg and 38 mg/kg in starter I and II phases, respectively), making it likely deficient relative to pig requirements and potentially contributing to the reduced growth performance of the NC-fed pigs. In contrast, levels of Cu, Fe, and Mn were close to, or above, NRC (2012) recommendations. Nevertheless, the bioavailability of these TM would have been low (Byrne and Murphy, 2022) so there may also have been some deficiency in these TM. Zinc plays a vital role in growth and development, regulating physiological processes and as a key-component and activator of enzymes. Zinc deficiency in weaned pigs leads to growth retardation, malnutrition, skin hyperkeratosis, and feed refusal (Tang et al., 2024). There was no marked suppression of feed intake or feed efficiency (gain: feed) in the unsupplemented NC diet but the tendency for reduced feed intake during the overall period likely contributed to the reduced weight gain in this group; weight gain is highly related to zinc intake in weaned pigs (Hansen et al., 2022).

Supplementation of TM to the NC improved weight gain and tended to increase feed intake over the 6-wk experiment. A stimulatory effect of supplemental Zn on feed intake is well described in young pigs (Hansen et al. 2022) which may operate through the stimulation of ghrelin production in the stomach fundus leading to a hunger response (De Mille et al., 2022) or via stimulation of the vagus nerve which promotes feeding behavior more widely in mammals (Ohinata et al., 2009). Supplemental Cu also stimulates feed intake in young pigs, through similar mechanisms (Villag**ó**mez-Estrade et al., 2020; De Mille et al., 2022) and may also have contributed here. Increased feed intake in the TM-supplemented pigs explains part of their improved weight gain (vs pigs fed the NC diet) but a greater inclusion level of bioavailable TM is reasoned to have been the major cause. Serum and liver tissue TM concentrations (indicators of functional TM status and of body stores of TM, respectively) were not increased in the TM-supplemented pigs but the increased utilization of Zn in bone in PC relative to the NC is a clear indication of greater bioavailable and absorbed Zn in the TM-supplemented pigs.

Supplementation of PhyG to the NC2 without added TM improved growth performance (weight gain and a tendency to increase feed intake). Given the documented mode of action of PhyG in pigs in improving the digestibility and utilization of macrominerals (Ca and P) by hydrolyzing phytate in the upper digestive tract (Christensen et al., 2020; Espinosa et al., 2021; Velayudhan et al., 2025), and the known affinity of TM cations to bind phytate in a similar manner to (or via) Ca, it is hypothesized that the improved weight gain in the PhyG-supplemented pigs was due to reduced TM-phytate binding which increased TM bioavailability. A similar effect was recently reported from the same PhyG in broilers (Dersjant-Li et al., 2025) and there is also some evidence of a similar effect in swine with other phytases. Shelton et al. (2005) observed improved weight gain from supplementation of an *Aspergillus niger* phytase (dosed at 500 FTU/kg) added to a diet containing no supplemental TM in young pigs, and with the same phytase supplemented at 1,350 U/g without added Zn, Lei et al. (1993) observed enhanced weight gain, feed intake and gain: feed in weanling pigs.

Other studies have observed that individual phytases can increase the digestibility of individual TM in pigs, including Zn (Ketata et al., 2024), Cu (Kies et al., 2005; She et al., 2018), and Fe (She et al., 2018). Digestibility was not measured in the present study, but tissue TM levels can provide additional information on TM bioavailability and utilization. Phytase did not result in any significant increase in the levels of Zn, Cu, Fe, Mn, or Se in the serum [but a numerical increase was observed for Zn (+22% vs NC1), Cu (+7.6% vs NC1), and Mn (24% vs NC1)] and there was no increase in the storage of these TM in the liver. However, there was a clear positive effect on Zn and Fe accumulation in bone. An explanation for the differential effect of the supplemental phytase across the TM could be that Cu and Mn levels in the tissues of pigs fed the NC were maintained at sufficient levels by their adequate availability in the basal diet, which was at or above NRC (2012) recommended levels, whereas for Zn, levels in the basal diet were deficient so the additional Zn released by PhyG may have been directed toward bone stores to meet bone requirements for Zn. Another reason for the apparent greater response of Zn to the phytase may be due to its higher affinity for binding with phytate in the pig digestive tract than the other TM (Champagne and Fisher, 1990; Philippi et al., 2023). A lack of effect of PhyG on Se tissue availability was not unexpected given that phytate binds with cations whereas Se exists in the digesta primarily as anions (e.g. as ${\text{SeO}}_{4}^{2-}$ or ${\text{SeO}}_{3}^{2-}$); the related PhyG study in broilers also did not observe a beneficial effect of PhyG on Se bioavailability (Dersjant-Li et al., 2025). The positive effect on bone Zn is consistent with the observations of Czech et al. (2022) for an Aspergillus *oryzae* phytase dosed at 250 to 1,500 FTU/kg in older (grower and finisher) pigs, in which no effect on plasma TM concentrations was observed but Zn and Cu were increased in bone ash. Meanwhile, in weaned pigs, Revy et al. (2006) observed increased liver and plasma Zn from supplementation of an *A. niger* phytase dosed at 700 FTU/kg and, in a separate study of the same phytase dosed at 1,200 FTU/kg (Revy et al., 2004), the authors observed an improvement in plasma Zn and liver Zn, Cu and Fe concentrations. Meanwhile, a recent meta-analysis of 24 publications covering pigs of all growth stages concluded that phytase improved the digestible Zn content of the diet but not the digestible Cu content (Ketata et al., 2024). Our findings support the existing literature reporting a consistent effect of phytase on Zn availability whereas its effect on the availability of the other TM (Cu, Fe, and Mn) was less certain, likely because of the higher levels of these TM in the basal diet. The differing results among studies regarding which tissues and which TM (beyond Zn) were affected may be linked to differences in the TM content of the basal diets and how TM deficient they were, the properties of the individual phytases, the phytase dose applied and the TM status of the animals prior to experimentation.

Multiple previous studies have observed a negative effect of the release of Zn by phytase (or equally of high levels of supplemental Zn) on Cu availability in young pigs (Cheng et al., 1998; Zacharias et al., 2003; Revy et al., 2004) but no such effect was observed here. The previous studies have reported reduced plasma or liver concentrations of Cu when phytase, supplemental Zn or both were added to the diet. The meta-analysis by Ketata et al. (2024) similarly observed an antagonism between the dietary concentration of Zn and the digestible content of Cu in the diet. This effect has been discussed as being due to stimulation of the synthesis of metallothioneins by Zn which bind Cu in the intestinal wall (Cousins, 1985) as well as direct competition between Cu and Zn at sites of absorption due to sharing of the same divalent metal transporter for uptake (Broom et al., 2021). In the present study, it may have been the case that the levels of available Zn in the Zn-unsupplemented, PhyG-supplemented diet, even with the beneficial activity of the PhyG in releasing Zn from phytate, was insufficient to cause detectable antagonization with Cu.

The PhyG phytase was more effective than TM supplementation (PC) at improving Fe and Zn levels (and Ca levels) in bone. The reason for this is unknown, but it could be that the Fe and Zn released from phytate by PhyG were more readily absorbed than the supplemental TM in the PC added as sulfate; it has been shown that TM added as sulfate are generally less readily absorbed in pigs than TM from other sources, especially organic or hydroxy forms (Ettle et al., 2008; Nielsen et al., 2022). The form of TM released by PhyG is currently unknown. The comparable (not significantly different) growth performance of pigs fed NC2+PhyG with that of those fed the PC, given that the basal NC2 diet contained only 40 to 44 mg/kg of Zn (analyzed values) which is well below the NRC (2012) recommendations of 100 mg Zn/kg in pigs of 7 to 11 kg and 80 mg Zn/kg in 11 to 25 kg pigs, suggests that the enzyme could support a reduction or even removal of supplemental Zn levels from the diet, dependent on the level of Zn in the basal diet. Such a reduction could serve to reduce Zn excretion and therefore Zn waste in pig production.

To the authors’ knowledge, this effect of a phytase totally replacing added TM in an all-vegetable diet has not been previously reported in young (weaned) pigs. The study by Shelton et al. (2005) observed that the added phytase prevented the reductions in growth performance that had been observed when TM were withdrawn from the diet but the basal diet contained fishmeal which would have provided an additional source of TM. However, it should be cautioned that these results were achieved in a research environment in which the number of pigs per pen was low (4) and therefore the pathogen challenge load was also likely to have been low. The capacity of the phytase to replace supplemental TM in a commercial field situation remains to be investigated. However, the results of this study demonstrated that PhyG phytase improved bioavailability of TM from basal ingredients and able to meet the nutritional requirement for bone mineralization and growth, this supports a reduction of added TM to the diet and will help to reduce the excretion of these TM to the environment.

## Conclusion

The stated hypothesis was upheld: the consensus bacterial 6-phytase variant (PhyG) supplemented at 1,500 to 1,000 FTU/kg to an all-vegetable, mixed-cereal diet without added TM, achieved growth performance responses and tissue TM concentrations comparable to those achieved by a nutritionally adequate diet supplemented with Zn, Cu, Fe, and Mn at 120, 80, 96, and 80 mg/kg, respectively, in weaned pigs of 9 to 25 kg BW. Overall, the findings suggest that: 1**)** in diets without PhyG, supplemented TM are needed to support optimal growth performance and bone mineralization in young pigs, and; 2**)** in diets containing PhyG (at a dose level of 1,500 and 1,000 FTU/kg in starter I and II phases, respectively), the enzyme could potentially be effective in totally replacing supplemental Zn, and reduction for the other TM (Cu, Fe, and Mn), without a negative effect on growth or bone mineralization.

## Abbreviations:

AA: amino acids
AAS: atopic absorption spectroscopy
ADFI: average daily feed intake
ADG: average daily gain
ANOVA: analysis of variance
ATTD: apparent total tract digestibility
BW: body weight
CF: crude fiber
CP: crude protein
DM: dry matter
FTU: phytase units
ICP-OES: inductively coupled plasma optical emission spectroscopy
n.a.,: not analyzed
NC: negative control
PC: positive control
PhyG: a consensus bacterial 6-phytase variant
SID: standardized ileal digestible
TM: trace minerals
